# Variation in Total Polyphenolics Contents of Aerial Parts of *Potentilla* Species and Their Anticariogenic Activity 

**DOI:** 10.3390/molecules15074639

**Published:** 2010-06-29

**Authors:** Michal Tomczyk, Malgorzata Pleszczyńska, Adrian Wiater

**Affiliations:** 1 Department of Pharmacognosy, Faculty of Pharmacy, Medical University of Białystok, ul. Mickiewicza 2a, 15-230 Białystok, Poland; 2 Department of Industrial Microbiology, Institute of Microbiology and Biotechnology, Maria Curie-Skłodowska University, ul. Akademicka 19, 20-033 Lublin, Poland; E-Mails: mplesz@poczta.onet.pl (M.P.); adrianw2@o2.pl (A.W.)

**Keywords:** *Potentilla*, Rosaceae, polyphenolic compounds, anticariogenic activity

## Abstract

The aerial parts of selected Potentilla species (*P. anserina, P. argentea, P. erecta, P. fruticosa, P. grandiflora, P. nepalensis, P. norvegica, P. pensylvanica, P. crantzii* and *P. thuringiaca*) were investigated in order to determine their contents of polyphenolic compounds. The results showed that *P. fruticosa* has relatively high concentrations of tannins (167.3 ± 2.0 mg/g dw), proanthocyanidins (4.6 ± 0.2 mg/g dw) and phenolic acids (16.4 ± 0.8 mg/g dw), as well as flavonoids (7.0 ± 1.1 mg/g dw), calculated as quercetin. Furthermore, we investigated the *in vitro* inhibitory effects of aqueous extracts from these species against cariogenic *Streptococcus* spp. strains. It was found that the tested samples moderately inhibit the growth of oral streptococci. However, all the preparations exhibited inhibitory effects on water-insoluble α-(1→3)-, α-(1→6)-linked glucan (mutan) and artificial dental plaque formation. The extract from *P. fruticosa* showed the highest anti-biofilm activities, with minimum mutan and biofilm inhibition concentrations of 6.25–25 and 50–100 µg/mL, respectively. The results indicate that the studied *Potentilla* species could be a potential plant material for extracting biologically active compounds, and could become a useful supplement for pharmaceutical products as a new anticariogenic agent in a wide range of oral care products.

## 1. Introduction

*Potentilla* species (Rosaceae) and their extracts have been highly valued in many different ethnic cultures for hundreds of years throughout the world. The genus *Potentilla* is well known for the difficulties it presents in terms of identifying species, as well as the frequency of synonyms which occur. A common phenomenon within this genus is interspecies hybridization and apomixis. This has resulted in a great morphological variability of species [1,2,3,4,5,6,7]. Extracts from *Potentilla* species were and are still applied for the treatment of inflammations, wounds, certain forms of cancer, infections due to bacteria, virus or fungi, diarrhoea, diabetes mellitus and several more ailments. The application of modern phytochemical methods have led to the isolation of a wide range of natural compounds from the various species. Above all tannins, e.g. condensed (proanthocyanidins) and hydrolysable tannins, are present in high amounts in both the aerial and the underground parts (roots, rhizomes) of *Potentilla *spp. Furthermore, a large number of triterpenoid structures have been isolated and elucidated, along with organic acids, numerous flavonoids, coumarins, polyprenols and other compounds. Most of the biological activities of *Potentilla *extracts can be explained by their high contents of polyphenolics. *Potentilla *extracts are presumed to be safe [8] and no toxic effects have been observed for them when applied to humans. However, further pharmacological studies are urgently needed in order to further prove the efficacy and potential benefit of these extracts. *Potentilla *extracts might become, or remain, important sources for herbal remedies as an alternative to chemically defined drugs, e.g. in antimicrobial therapy [9,10,11].

In the recent past, there has been an increased interest in the therapeutic properties of some medicinal plants and natural compounds which have demonstrated anticariogenic activities *in vitro* and *in vivo*. Among these phytoconstituents, several polyphenolic compounds like tannins (catechins) and flavonoids seem to be the most promising biomolecules. Remarkable anticariogenic potency has also been observed for alkaloids [12,13,14,15]. Bioactive constituents play a very important role in multiple mechanisms, which may be responsible for many pharmacological effects. Numerous synthetic substances and antibiotics have been used in the control of dental plaque. However, these compounds cause many unexpected side-effects. It has been claimed that the use of medicinal plant and natural products, incorporated in food and beverages (juices, fruits), offers potential candidates for anticariogenic agents [15,16,17]. Considering the traditional use of the *Potentilla* species, it would be meaningful to investigate its phytochemical profile and biological properties in the dental field for the first time. 

The purpose of our study was to evaluate the effects of aqueous extracts from the aerial parts of selected *Potentilla* species against cariogenic *Streptococcus* spp. strains. Furthermore, their inhibitory effects on water-insoluble α-(1→3)-, α-(1→6)-linked glucan (mutan) and artificial dental plaque formation were examined. The phytochemical profile of the active constituents: phenolic acids, flavonoids and tannins, as well as total polyphenol content in the investigated samples were also analyzed. 

## 2. Results and Discussion

### 2.1. Determination of polyphenolic compounds

The total polyphenol content, and that of related polyphenolic compounds (phenolic acids, flavonoids, tannins, proanthocyanidins) in the aerial parts of selected *Potentilla* species (Table 1) were measured by different analytical methods.

**Table 1:** Plant material from the selected *Potentilla* L. species.

Sample no.	Plant sources		Parts used	Voucher specimen no.
	Scientific name	Common name		
1.	*Potentilla argentea *L.	silver cinquefoil	herbs	PAR-02009
2.	*Potentilla erecta *L.	erect cinquefoil	herbs	PER-06016
3.	*Potentilla anserina *L.	silverweed cinquefoil	herbs	PAN-06017
4.	*Potentilla fruticosa *L.	shrubby cinquefoil	herbs	PFR-06018
5.	*Potentilla grandiflora *L.	cinquefoil*	herbs	PGR-06020
6.	*Potentilla norvegica* L.	norwegian cinquefoil	herbs	PNO-08024
7.	*Potentilla thuringiaca* Bernh. ex Link	european cinquefoil	herbs	PTH-06022
8.	*Potentilla pensylvanica* L.	pennsylvania cinquefoil	herbs	PPS-08025
9.	*Potentilla crantzii *(Crantz) J. Beck ex Fritsch	alpine cinquefoil	herbs	PCR-09026
10.	*Potentilla nepalensis* Hook. var. ‘Miss Willmott’	nepal cinquefoil	herbs	PNE-06023

* no common name.

The total polyphenol content in aqueous extracts were determined from the regression equation of the calibration curve and expressed in gallic acid equivalents (GAE). The content of total polyphenols ranged from 49.9 ± 1.5 mg GAE/g dw for the aerial parts of *P. pensylvanica* to 116.3 ± 3.9 mg GAE/g dw for *P. fruticosa.* All these differences in the values of total phenolic content in all the analyzed fractions and extracts can be attributed to the differences in the composition of these ten extracts. It was obvious that the total polyphenolic compounds (TPC) determined by Folin-Ciocalteu’s method had not given a full characterisation of the quality and quantity of the various groups of polyphenolic compounds. The presence of these groups of compounds encouraged us to determine them in plant material by the use of other methods (weight and spectrophotometrical). Our data (Table 2) show that the aerial parts of *P. fruticosa* have relatively high concentrations of tannins (167.3 ± 2.0 mg/g dw), proanthocyanidins (4.6 ± 0.2 mg/g dw), and phenolic acids (16.4 ± 0.8 mg/g dw), as well as flavonoid compounds (7.0 ± 1.1 mg/g dw), calculated as quercetin units. In most of the samples, the values obtained in our work for total tannin content (TTC) in all the analysed extracts were higher that those reported as the sum of all polyphenolic compounds (TPC). The significant differences found between those values arise from the use of two different analytical methods. In our case, the total tannin content (TTC) was determined by using the imprecise weight method with hide powder. This method, based on protein precipitation, is often cited as being more realistic for estimating the total content of tannins in plant material because the method is more closely related to biological effect. The method, which relies on the precipitation of protein from solution, usually determines the amount of protein precipitated, or the amount of phenolic compounds that remain in solution [18].

**Table 2:** Extraction yield, total phenolic (TPC), total phenolic acids (TPA), total flavonoids (TFC), total tannins (TTC) and total proanthocyanidins (TPDC) content of *Potentilla* species.

Sample no. ^a^	Yield (wt %)	Polyphenols content (mg/g dw) ^b^				
		TPC	TPA	TFC	TTC	TPDC
1.	6.80	57.4 ± 0.8	6.2 ± 0.3	1.9 ± 0.7	87.5 ± 2.4	2.5 ± 0.3
2.	5.15	69.3 ± 1.4	3.7 ± 0.5	2.5 ± 0.3	71.3 ± 1.8	3.8 ± 0.2
3.	4.90	89.8 ± 2.1	5.6 ± 0.2	4.9 ± 0.2	95.2 ± 2.2	4.2 ± 0.4
4.	3.45	116.3 ± 3.9	16.4 ± 0.8	7.0 ± 1.1	167.3 ± 2.0	4.6 ± 0.2
5.	4.65	71.1 ± 5.7	4.1 ± 0.4	2.7 ± 0.2	98.7 ± 2.2	3.9 ± 0.4
6.	3.75	82.9 ± 2.2	8.5 ± 0.6	2.8 ± 0.6	72.1 ± 0.8	4.3 ± 0.1
7.	5.85	58.4 ± 3.1	4.5 ± 0.2	5.1 ± 0.3	72.5 ± 1.2	4.1 ± 0.2
8.	3.10	49.9 ± 1.5	6.8 ± 0.5	5.5 ± 0.3	59.7 ± 2.1	3.3 ± 0.2
9.	2.85	94.8 ± 2.8	5.3 ± 0.2	4.8 ± 0.4	72.2 ± 0.9	4.5 ± 0.3
10.	2.11	73.9 ± 3.7	7.7 ± 0.4	2.1 ± 0.5	65.7 ± 1.9	3.7 ± 0.1

^a ^Numbering of samples (extracts) is equal to numbering of analyzed species (see Table 1); ^b^ Results are means ± SD of three different experiments (n = 3)

The aerial parts of *P. fruticosa* have been studied extensively and have been found to contain a wide variety of polyphenolics, such as ellagic acid, catechins and flavonols (quercetin, kaempferol and rhamnetin glycosides) [19,20,21,22,23,24]. Our results show that *P. fructicosa *is rich in polyphenolic compounds, and they are consistent with earlier studies on reported by Shikov *et al. *[19] and Miliauskas *et al*. [20]. 

Some of the polyphenolic compounds isolated from plants exhibit anticaries activity, due either to growth inhibition against oral bacteria, or to the inhibition of glucosyltransferases (GTF) activity. In previous studies it was shown that hydrolysable tannins (gallo- and ellagitannins) from crude drugs were more potent that chlorhexidine in aqueous solution [25]. Similar results were observed in the study on pure condensed tannins (proanthocyanidins), (-)-epigallocatechin gallate and (-)-epicatechin gallate, which showed the most potent inhibition [26]. Furthermore, Duarte *et al*. proved that the inhibitory effects of proanthocyanidin and flavonoid fractions, alone or in combination, as well as crude extracts from cranberry fruits (*Vaccinium macrocarpon* Ait., Ericaeae), reduce the formation and accumulation of *S. mutans* biofilms by mostly diminishing the amounts of insoluble glucans in the biofilms matrix. Additionally, the effective inhibition of GTFs was also observed in their study. They also suggested that the biological activity of cranberry extracts resulted from a complex of polyphenolics rather than a single active constituent [27]. Earlier studies with various apple polyphenols indicated that most low-molecular-weight polyphenols do not inhibit GTF activity, except for some aglycone-form compounds (such as flavonoids: quercetin and phloretin) and galloyl ester derivatives of (-)-epigallocatechin gallate or ellagic acid, but in contrast, high-molecular-weight polyphenolics (flavan-3-ol and oligomers) showed strong inhibitory action [17].

### 2.2. Anticariogenic activity

All the tested aqueous extracts possess a mild antibacterial activity. Only the extract from *P. fruticosa *effectively inhibited the growth of oral bacteria; the minimum inhibitory concentrations were 3.2 mg/mL against all test streptococci. The same concentrations killed 99.9% of the test bacteria. The antibacterial property of this extract is suspected to be associated with the high concentrations of polyphenolic compounds, some of which are known to possess strong antibacterial activity [28,29]. 

It has been well established that the main etiological factor of dental caries and periodontal disease is dental plaque. The control of dental plaque has been a target in dental caries prevention. The glucosyltransferases (GTFs) are critical in the synthesis of extracellular polysaccharides (EPS) from sucrose. EPS (especially mutan) promote the selective adherence and accumulation of mutans streptococci on the teeth and increase the bulk and porosity of dental plaque [30]. Our study, for the first time, demonstrated that aqueous extracts from *Potentilla* species are potent inhibitors of the enzymatic activity of GTF enzymes (mutan and biofilm formation). The MMIC_50_ and MBIC_50_ values of *Potentilla* species extracts are listed in Table 3. All ten tested extracts effectively suppressed insoluble glucan synthesis and mutans streptococci biofilm formation. However, a preparation with higher concentrations of polyphenols derived from *P. fruticosa* clearly showed the strongest inhibitory effect (MMIC_50 _from 6.25 to 25 μg/mL, MBIC_50_ from 50 to 100 μg/mL). The weakest effects were observed with *P. thuringiaca* extract (MMIC_50 _from 50 to 200 μg/mL, MBIC_50 _400 μg/mL). 

**Table 3:** Effects of *Potentilla *species aqueous extracts on the mutan synthesis (MMIC_50_) ^a^ and on the formation of mutans streptococci biofilm (MBIC_50_) ^b^.

Sample no./Control	MMIC_50_ (μg/mL)^a^			MBIC_50_ (μg/mL) ^b^		
	*S. mutans* 6067	*S. sobrinus* 6070	*S. sobrinus*/*downei* 21020	*S. mutans* 6067	*S. sobrinus* 6070	*S. sobrinus*/*downei* 21020
1.	100	25	100	100	100	200
2.	25	25	100	400	400	400
3.	50	25	100	100	100	200
4.	6.25	6.25	25	50	100	50
5.	100	25	100	100	100	100
6.	25	50	50	100	100	100
7.	50	100	200	400	400	400
8.	25	50	100	200	200	400
9.	50	50	100	50	100	100
10.	50	50	50	100	100	50
Chlorhexidine	-	-	-	0.5	0.5	0.5
Ellagic acid	1.87	1.87	3.75	-	-	-

^a^ MMIC_50_ – minimum mutan inhibition concentration, the lowest agent concentration that showed >50% inhibition on mutan synthesis; ^b^MBIC_50_ – minimum biofilm inhibition concentration, the lowest agent concentration that showed > 50% inhibition on biofilm formation.

The main biologically active compounds in the tested extracts are polyphenols. The health benefits of these compounds include antioxidant and anticancer, as well as antibacterial and anti-inflammatory properties. The results of the anticariogenic activity for the individual constituents were not as good as the polyphenolic complex, suggesting that there may be a synergistic effects between these compounds [31]. It is probable that polyphenol fractions bind to and/or mask hydrophobic proteins on the cell surface of oral bacteria [32]. Many studies have been attempts to use natural extracts, derived from plant material, for anticariogenic activity. Green tea [33], oolong tea [34], propolis [35], garlic [36], cocoa bean [37], *Humulus lupulus* [38] and *Galla chinensis* [39] have been reported to significantly inhibit the growth of oral bacteria and to possess a powerful anti-GTF activity, as well as reducing the cell surface hydrophobicity of oral streptococci. This latter factor enables bacteria to adhere to the tooth surface. They also showed a reduction in acid production in mutans streptococci [40,41,42]. The significant inhibition of mutan synthesis and biofilm formation demonstrated by various *Potentilla* species extracts shows them to be promising natural products for the prevention of dental caries.

## 3. Experimental

### 3.1. Materials and methods

#### 3.1.1. Chemicals and analytical instruments

All solvents of analytical grade were purchased from POCH, S.A. (Gliwice, Poland). Hide powder was purchased from Fluka (Buchs, Switzerland). Milli-Q Plus (Millipore, MA, USA) treated water (18.2 MΩ cm) was used throughout the analysis. The absorbances were measured on a Specord 40 UV-VIS instrument (Jena Analytik AG, Germany).

### 3.2. Plant material

Seeds of seven species (*P. fruticosa – *index seminum /ind. sem./ 2566, *P. grandiflora *– ind. sem. 758, *P. norvegica* – ind. sem. 303,* P. thuringiaca* – ind. sem. 1551, *P. pensylvanica *– ind. sem. 1546*, P. crantzii *– ind. sem. 1534*, P. nepalensis* – ind. sem. - 1542) were obtained from the Hortus Botanicus Universitatis Masarykianae, Brno, Botanical Garden of Vilnius University, Lithuania, Czech Republic, Hortus Botanicus Universitatis Posnaniensis, Poznań, Polska and the Giardino Botanico Alpino, Cogne, Italy. Plants were cultivated in a common plot at the Medicinal Plants Garden near Medical University of Białystok, Poland. Aerial parts of *P. anserina, P. argentea *and *P. erecta *were collected from Puszcza Knyszyńska, near Białystok, Poland. Voucher specimens of plants have been deposited in the Herbarium of the Department of Pharmacognosy, Medical University of Białystok, Poland. Collected plant materials (aerial parts), during June-July 2005-2009, were air-dried under shade at room temperature and then ground with an electric grinder into fine powders which were stored into airtight containers at room temperature. The list of studied plants is given in Table 1.

### 3.3. Sample extraction

Powdered plant material (2.0 g) was separately extracted with water (2 × 150 mL) in an ultrasonicator bath (Sonic-5, Polsonic, Poland) at controlled temperature (40 ± 2 °C) for 45 min. Supernatants were filtered through a funnel with glass wool, which was washed with solvent (5 mL) and concentrated to dryness under vacuum (Büchi System, Switzerland) at controlled temperature (40 ± 2 °C) and subjected to lyophilization using a Lymph-Lock 1.0 (Labconco, USA) vacuum concentrator until a constant weight were obtained. The list of analyzed extracts is given in Table 1. 

### 3.4. Phytochemical profile

#### 3.4.1. Determination of total polyphenolic content

Total polyphenolic contents in extracts were determinated spectrophotometrically at 765 nm (Specord 40, Analytik Jena, Germany) after the reaction with Folin-Ciocalteu’s phenol reagent as gallic acid equivalents GAE/100g in mg per g of dry weight (dw) according to the manual colorimetric method described by Tawaha [43]. A sample aliquot of extract and subfraction (50 μL) were dissolved in distilled water (450 μL) and 0.2 N Folin-Ciocalteu’s reagent (2.5 mL). After 5 min saturated sodium carbonate Na_2_CO_3 _solution (2 mL, 75 g/L) were added. Samples were vortexed and incubated in the dark at room temperature for 2 h. Quantitative measurements were performed, based on a standard calibration curve of different concentrations of gallic acid (20–500 mg/L). All measurements were performed in triplicate. The results are given in Table 2.

#### 3.4.2. Determination of total phenolic acids content

Total phenolic acids content in plant material was determined using the spectrophotometric method with Arnov’s reagent according to the procedure described in the Polish Pharmacopoeia VI [44]. Stock solution was prepared from the powdered sample (1.0 g) mixed with water (25 mL, two times) and shaken for 30 min each, then filtered. Phenolic acids were determined from an aliquot of stock solution (1 mL) mixed with water (5 mL), hydrochloric acid (1 mL, 18 g/L), Arnov’s reagent (1 mL) and of sodium hydroxide solution (1 mL, 40 g/L) and diluted with water to 10 mL. Phenolic acids were measured spectrophotometrically at 490 nm The percentage of phenolic acids, expressed as caffeic acid equivalent on dry weight, is calculated from the expression:

*A* × 1.7544/*m*

where *A* is the absorbance of the test solution at 490 nm and *m* mass of the powdered drug, in grams. The results are given in Table 2 and are means of experiments conducted in triplicate.

#### 3.4.3. Determination of total flavonoid content

The total content of flavonoids was determined by the spectrophotometric method of Christ and Müller [45] and followed the procedure described in the Polish Pharmacopoeia VI [44]. Each powdered sample (0.6 g) was mixed with acetone (20 mL), 25% hydrochloric acid (2 mL, 281 g/L) and 0.5% urotropine (methenamine) solution (1 mL, 5 g/L) and heated in a water bath under reflux for 30 min. The obtained extract was filtered through cotton wool, and the sediment with the cotton wool was heated twice for 10 min. with acetone (20 mL). The extracts were mixed and diluted with acetone to 100 mL in a volumetric flask. Then, a portion of this solution (20 mL) was diluted with water (20 mL), extracted with ethyl acetate (15 mL) and then three times more with ethyl acetate (10 mL). Organic phases were mixed and washed twice with water (40 mL), fitered into volumetric flasks and diluted with ethyl acetate to 50 mL. The obtained solution (10 mL) was added to four volumetric flasks of. Then, to three flasks aluminium chloride (2 mL, 20 g/L – methanolic solution) was added and all four flask were made up to 25 mL with methanol - acetic aid glacial (19:1). After hydrolysis, the flavonoids were measured spectrophotometrically at 425 nm by creating a complex with aluminium chloride in a methanol–ethyl acetate–acetic acid medium. The contents of total flavonoids, expressed as quercetin equivalent on dry weight, were calculated from the expression, respectively:

*A* × 0.875/*b*

where *A* is the absorbance of the test solution at 425 nm and *b* the mass of the powdered drug, in grams. The results are given in Table 2.

#### 3.4.4. Determination of total tannin content

The total tannin content was determined by the weight method with hide powder according to the DAB 10 [46]. The results are given in Table 2.

#### 3.4.5. Determination of total proanthocyanidin content

The total proantocyanidin content was measured according to the European Pharmacopoeia [47]. Accurately weighed plant material (2.5 g) was heated under reflux for 30 min with ethanol (30 mL, 70% v/v). After that extract was filtered and the residue was flashed with ethanol (10 mL, 70% v/v). Then 25% hydrochloric acid (15 mL) was added with water (10 mL). The solution was heated under reflux for 80 min. After cooling, the extract was filtered and made up with ethanol (70% v/v) to 250 mL. Then 50 mL od solution was evaporated to about 3 mL and transferred to a separating funnel with water (15 mL). The solution were then extracted with *n*-butanol (3 × 15 mL, each). The organic layers were mixed and transferred to volumetric flasks and made up with *n*-butanol to 100 mL. The absorbance was measured spectrophotometrically at 545 nm. The contents of proanthocyanidin, expressed as cyanidin chloride equivalent on dry weight, were calculated from the expression:

*A* × 500/75 × *m*

where *A* is the absorbance of the test solution at 545 nm and *m* the mass of the powdered drug, in grams, respectively. The results are given in Table 2.

### 3.5. Anticariogenic activity

#### 3.5.1. Bacterial strains, media and growth conditions

The cariogenic streptococci used in this study included *Streptococcus mutans* CAPM 6067, *S. sobrinus* CAPM 6070 (The Collection of Animal Pathogenic Microorganisms, Brno, Czech Republic), and *S. sobrinus*/*downei* CCUG 21020 (formerly *S. mutans* OMZ 176) (The Culture Collection, University of Göteborg, Sweden). The strains were stored as glycerol stocks at −20 °C. Bacteria were grown at 37 °C in minimum medium for *S. mutans* (MM medium) [48] containing (g/L) glucose (10), L-glutamic acid (2), L-cysteine-HCl (0.2), L-leucine (0.9), NH_4_Cl (1), K_2_HPO_4_ (3.5), KH_2_PO_4_ (1.5), NaHCO_3_ (4.2), MgSO_4_·7H_2_O (1.2), MnCl_4_·4H_2_O (0.02), FeSO_4_·7H_2_O (0.02), sodium pyruvate (0.6), and (mg/L) riboflavin (1), thiamine-HCl (0.5), biotin (0.1), nicotinic acid (1), *p*-aminobenzoic acid (0.1), calcium pantothenate (0.5), pyridoxal·HCl (1), tris-maleate buffer (pH 7.4) to 1 L. The final pH of the medium was 7.1. After 24 h incubation, bacterial cultures were centrifuged, and the supernatants were used as cell-free glucosyltransferases.

#### 3.5.2. Determination of minimum inhibitory and minimum bactericidal concentrations

The aqueous extracts from aerial parts of selected *Potentilla* species were tested for antibacterial activity by the broth dilution method. The minimum inhibitory concentrations were determined in 96-well cell culture plates (Nunc, Denmark), and were carried out in triplicate. A two-fold serial dilution of each extract in 1% DMSO was prepared. Plates with wells containing MM medium (160 μL) plus 1% DMSO (40 μL), 100% growth controls) and MM medium (160 μL) plus each dilution of plant extracts (40 μL, final concentration from 6.4 to 0.006 mg/mL) were inoculated with 10^5^–10^6^ test bacteria, and cultured for 1–2 days at 37 °C. The minimum inhibitory concentrations (MICs) were determined as the lowest concentration of test samples that resulted in a complete inhibition of visible growth in the broth. After the measurement of MICs, 10-μL aliquots of cultures were taken from wells showing no growth, inoculated into BHI agar plates, and cultured for 1 week. The minimum bactericidal concentrations (MBCs) were determined on the basis of the lowest concentration of the test extracts that kills 99.9% of the test bacteria. Chlorhexidine digluconate (Sigma-Aldrich, Germany) was used as a standard antibiotic in order to compare the sensitivity of the extracts against test bacteria.

#### 3.5.3. Inhibition of mutan synthesis

The inhibitory effect of plant extracts on the mutan synthesis was examined. A two-fold serial dilution of each extract (concentration ranging from 6.25 to 800 μg/mL) in 1% DMSO (100 μL final volume) was prepared in Eppendorf tubes. All tubes were filled with 24-h-old post-culture supernatant of the tested bacterium (1.0 mL) and sucrose solution (100 μL, final concentration of 3%, w/v) in 0.1 M potassium phosphate buffer (pH 6.0) in the presence of 0.05% sodium azide as a preservative. Ellagic acid (Sigma-Aldrich, Germany), in form of sodium ellagate, from 1.87 to 6.25 μg/mL, was used as the positive control, the medium without extracts was used as the non-treated control. After incubation for 24 h at 37 °C, the formed water-insoluble polysaccharide (mutan) was collected by centrifugation at 16099 x g for 10 min, washed thoroughly with deionised water and determined, utilizing phenol-sulphuric acid method with glucose as a standard [49]. The minimum mutan inhibition concentration (MMIC_50_) was defined as the lowest agent concentration that showed 50% or more inhibition on the mutan synthesis. 

#### 3.5.4. Inhibition of biofilm formation

The effect of plant extracts on the formation of cariogenic streptococci biofilm was examined according to a modification of the method described by Xiao *et al*. [50]. Serial dilutions of each extract in 1% DMSO were prepared. Aliquots (40 μL) of each dilution were dispensed in 96-well cell culture plates. Subsequently, portions of MM medium with 3% (w/v) sucrose (160 μL) were added, and 10^5^–10^6^ test bacteria were inoculated into each well. Final concentrations of extracts ranged from 6.25 to 800 μg/mL. Chlorhexidine was used as the negative control and the medium without extracts was used as the non-treated control. After incubation for 24 h at 37 °C, media and unattached to substratum cells were decanted and planktonic cells were removed by washing with PBS, pH 7.2. The adhered biofilm was air dried and stained (5 min) with 0.1% (w/v) Crystal Violet (Sigma-Aldrich, Germany) until control wells appeared colourless. Biofilm formation was quantified by the addition of 95% ethanol (300 μL) to each stained well. The plates were incubated for 24 h at room temperature and the adsorbance at 595 nm was determined using a microplate reader (Molecular Devices, USA). The percentage of inhibition was calculated using the equation:

(1 − A_595 _of the test/ A_595 _of non-treated control) × 100%

The minimum biofilm inhibition concentration (MBIC_50_) was defined as the lowest agent concentration that showed 50% or more inhibition on the formation of biofilm. 

## 4. Conclusions

The results of the study suggest that aqueous extracts from selected *Potentilla* species, especially, *P. fruticosa* extract, contain high concentrations of polyphenols such as tannins (proanthocyanidins) and phenolic acids, as well as flavonoids which can prevent dental caries, since they demonstrated antimicrobial activity against mutans streptococci and also inhibition of dental plaque formation *in vitro*. A relationship was also established between total polyphenol, flavonoid, phenolic acid and tannin as well proanthocyanidins content and anticariogenic activity, and it was observed that this activity increased proportionally with the polyphenolic content in the studied samples.
